# Patient-Reported Outcome and Experience Measures (PROMs and PREMs) in Pressure Injury Prevention and Care Across European Hospital and Community Nursing Settings: A Scoping Review

**DOI:** 10.7759/cureus.113636

**Published:** 2026-07-30

**Authors:** Vincenzo Abbasciano, Giuseppe Fumai, Giorgia Chetta, Gianluca Laricchia, Verdiana La Grotta, Giovanna Muto, Giuseppe Gisario, Carla Recchia, Nunzia Cappucci, Valeria Liso, Anna Maria Carrozzo

**Affiliations:** 1 Department of Internal Medicine, Hospital, Local Health Authority Barletta-Andria-Trani, Andria, ITA; 2 Biomedical Sciences, University of Foggia, Foggia, ITA; 3 Orthopedic and Neurological Rehabilitation, Rehcura Rehabilitation Facility, Adelfia, ITA; 4 Stakeholder Engagement and Innovation Governance, Agenzia Regionale Sanitaria della Puglia, Bari, ITA; 5 Nursing, University of Foggia, Foggia, ITA; 6 Information, Communication, University Liaison, and Training, Local Health Authority of Barletta Andria Trani, Barletta, ITA; 7 Cardiothoracic Surgery, University of Padua, Padua, ITA; 8 Emergency, Local Health Authority Foggia, Cerignola, ITA; 9 Complex Administrative-Legal Framework, Local Health Authority Foggia, Foggia, ITA; 10 Department of Orthopedics and Traumatology, Local Health Authority Barletta-Andria-Trani, Andria, ITA; 11 Local Health Authority, University of Foggia, Foggia, ITA

**Keywords:** community nursing, epuap/npiap staging, family and community nurse, intensive care, patient-reported experience measures, patient-reported outcome measures, pressure injury, pressure ulcer, scoping review

## Abstract

Pressure injuries are a frequent and largely preventable adverse event across the whole care continuum, from intensive and acute hospital wards to community and home settings. Internationally, they are graded with the objective European Pressure Ulcer Advisory Panel and National Pressure Injury Advisory Panel (EPUAP/NPIAP) staging system. Staging describes the tissue rather than the person. How patients experience each stage is captured by patient-reported outcome measures (PROMs), and how they experience prevention is captured by patient-reported experience measures (PREMs). Both may be recorded inconsistently and remain disconnected from the objective stage. Prevention and care are increasingly delivered by community and family nurses, yet the patient-reported axis in these settings, and in the newly established community hospitals and community houses, remains under-mapped.

This scoping review maps which PROMs and PREMs have been used for pressure injury prevention and care across European hospital and community nursing settings. It identifies which domains are captured at each EPUAP/NPIAP stage and during prevention, characterises the role of the community and family nurse and examines where dedicated protocols may be absent in community hospitals and community houses. The review was conducted following the updated Joanna Briggs Institute (JBI) methodology and reported using the Preferred Reporting Items for Systematic Reviews and Meta-Analyses Extension for Scoping Reviews (PRISMA-ScR). Three databases, namely, PubMed, Scopus and the Cumulative Index to Nursing and Allied Health Literature (CINAHL), were searched within a Population, Concept, Context (PCC) framework.

The searches yielded 891 records; after de-duplication, 666 records were screened, and 29 sources were included, of which 25 were conducted in European settings and 2 in Turkey, and 2 were multinational reviews. The instruments identified were the generic EuroQol five-dimension (EQ-5D) and 36-Item Short Form (SF-36) health surveys and the pressure ulcer-specific Pressure Ulcer Quality of Life (PU-QOL) and Wound Quality of Life (Wound-QoL) measures, together with the Quality from the Patient's Perspective (QPP) experience measure and qualitative interviews. The domains captured were pain, mobility and function, sleep, exudate and odour, dignity, psychological impact and health-related quality of life, alongside the experience domains of involvement, communication, continuity and satisfaction. Evidence was concentrated in the United Kingdom, Nordic countries, Switzerland, Spain and Italy, and community and district nursing were well represented.

Across these settings, the patient-reported axis was captured with a small and heterogeneous set of instruments and qualitative methods, and it was rarely linked to a specific EPUAP/NPIAP stage. No included source addressed the Italian community hospitals or community houses, and dedicated pressure injury protocols with standardised patient-reported measurement in these new intermediate care structures were not described. Stage-aware PROM and PREM instruments validated for critically ill and proxy respondents and for community use emerge as a priority for future work.

## Introduction and background

Pressure injuries are among the most frequent and largely preventable adverse events in healthcare, and they occur across the entire care continuum, from intensive and acute hospital wards to community, intermediate care and home settings. In a recent European national benchmarking study, hospital-acquired pressure ulcers were most frequent in intensive care units (ICUs), yet they remained a substantial burden in general wards and in community and home care [1]. Internationally, these lesions are graded with the objective European Pressure Ulcer Advisory Panel and National Pressure Injury Advisory Panel (EPUAP/NPIAP) staging system. The system was revised in 2016 to adopt the term injury and Arabic numerals, and it defines stage 1-4, unstageable and deep tissue pressure injury by the extent of tissue loss [2]. It is embedded in the EPUAP/NPIAP/Pan Pacific Pressure Injury Alliance (PPPIA) international clinical practice guideline, which spans acute, community and home settings [3].

Staging describes the tissue, not the person. Pressure injuries are nonetheless lived experiences. They cause pain, exudate, sleep disruption, restricted mobility, threats to dignity and psychological distress, and they impose a substantial burden on health-related quality of life in acute, community and long-term care [4,5]. A systematic review of outcome measures for chronic wounds found that no pressure injury-specific patient-reported outcome measure (PROM) adequately captured the domains that matter to patients [6]. The patient's voice is therefore both clinically consequential and methodologically under-served.

Two complementary families of patient-centred instruments are relevant. PROMs capture how patients feel and function, and in this context, how they experience each stage of injury. Patient-reported experience measures (PREMs) capture how patients experience the process of care, and in this context, the prevention of injury, including involvement in decisions, communication, the perception of repositioning and support surfaces, and satisfaction with preventive care [7,8]. PROMs and PREMs are increasingly recognised as core components of quality measurement, although their uptake is uneven across settings and clinical areas [7,8].

Pressure injury prevention and care are no longer confined to hospitals. Across Europe, prevention, skin assessment, repositioning, support surface management and the care of at-risk patients are increasingly delivered by community and family nurses. In Italy, the Family and Community Nurse (Infermiere di Famiglia e Comunità) was established by Law 77/2020 and Ministerial Decree (DM) 77/2022. This role has strengthened primary care, although with heterogeneous regional implementation and still-evolving activity catalogues [9]. DM 77/2022 also introduced new intermediate care structures, the community hospitals and community houses as the organisational backbone of territorial care [10]. Comparable community, district and public health nursing roles deliver pressure injury prevention and care abroad. Because these structures and roles are recent and heterogeneously implemented, dedicated protocols for the assessment, prevention and management of patients with pressure injuries in community hospitals and community houses may not yet be established. This review is designed to examine that potential evidence and practice gap.

Both ends of the continuum challenge the capture of the patient-reported axis, for different reasons. In high-acuity hospital care, patients are frequently sedated, ventilated or delirious, so direct self-report is often impossible and may depend on validated proxies. In community and home care, self-report is more feasible but is rarely standardised, and the experience of prevention reported by patients and families is seldom measured. The integrative question is therefore whether, and how, the literature connects these two patient-centred axes, the lived experience of the injury and the lived experience of its prevention, to the objective stage of tissue damage. This question spans hospital and community settings and the professionals, including the community and family nurse, who deliver that care.

To our knowledge, no scoping review has mapped the PROMs and PREMs used across the EPUAP/NPIAP staging continuum in European hospital and community nursing settings. Nor has any identified where the patient-reported axis, and dedicated protocols, may be absent, particularly in the new community structures. Because the aim is to map the breadth and nature of the evidence and to identify gaps rather than to estimate effect sizes, a scoping review is the appropriate design [11,12].

Framed with the Population-Concept-Context (PCC) mnemonic, detailed in the Methods, the review addresses two levels of question: a broad European question (which PROMs and PREMs are used across the EPUAP/NPIAP staging continuum in European hospital and community nursing, which domains they capture and whether the literature links these patient-reported axes to the objective stage) and a specific Italian sub-question (whether dedicated pressure injury protocols and standardised patient-reported measurement yet exist in the DM 77/2022 community hospitals and community houses and for the Family and Community Nurse who staffs them). The objectives are correspondingly to map the PROMs and PREMs used across these settings, to identify the domains captured during prevention and at each stage, to characterise the role of the community and family nurse and to identify any absence of dedicated protocols in the new community structures.

## Review

Methods

Study Design

This scoping review was conducted in accordance with the updated Joanna Briggs Institute (JBI) methodological guidance for scoping reviews [12,13], which builds on the framework proposed by Arksey and O'Malley [14] and its refinement by Levac et al. [15]. It was reported in accordance with the Preferred Reporting Items for Systematic Reviews and Meta-Analyses Extension for Scoping Reviews (PRISMA-ScR) checklist [16] and the PRISMA 2020 statement [17]. In accordance with JBI guidance, no formal quality appraisal of included sources was performed, because the purpose of a scoping review is to map the extent and nature of the available evidence rather than to produce a weighted synthesis [12].

This scoping review was not registered in a prospective registry. PROSPERO does not accept scoping review protocols, and prospective registration is not a requirement for scoping reviews; the review followed an a priori plan defined by the Population-Concept-Context framework and the eligibility criteria reported here.

Search Strategy

Three bibliographic databases were searched, namely, PubMed (MEDLINE), Scopus and Cumulative Index to Nursing and Allied Health Literature (CINAHL) Complete (via EBSCOhost). An explicit publication date limit of 2014-2025 was applied to PubMed and Scopus, anchored to the 2014 international pressure ulcer guideline. The CINAHL search, the principal nursing database for this review, was deliberately not year-restricted, in order to capture the foundational nursing literature on health-related quality of life in pressure injury that predates the guideline; this is consistent with the CINAHL string reported in Table 1, which carries no publication year filter. Two included sources carried a 2026 issue date but were early-access publications already available and indexed within the search window.

**Table 1:** Boolean search strings used in each database Publication year and language limits are embedded as executable syntax where the interface supports it, namely, PubMed with (english[la] OR italian[la]) and 2014:2025[dp] and Scopus with PUBYEAR > 2013 AND PUBYEAR < 2026. The PubMed Adult (19+) age limit is applied through the Ages filter, and the CINAHL, language and peer-reviewed limits through the EBSCOhost limiters; a publication year limit was not applied in CINAHL, so that seminal pre-2014 nursing sources on quality of life in pressure injury could be captured. Each cell holds only the query to be run; the descriptive limits are documented here and are not typed into the search box. The CINAHL search was restricted to the CINAHL database. The community and family nurse role and the dedicated protocol dimension are captured at the charting stage, and European origin is analysed as a lens. Per-database yields are reported in the Results after de-duplication of the exports. MeSH: Medical Subject Headings, MH: CINAHL Subject Heading (the + symbol explodes the heading), tiab: title/abstract, la: language, dp: date of publication, TITLE-ABS-KEY: Scopus title, abstract and keyword field, ICU: intensive care unit

Database	Search string
PubMed (MEDLINE)	(("Pressure Ulcer"[Mesh] OR "pressure ulcer*"[tiab] OR "pressure injur*"[tiab] OR "pressure sore*"[tiab] OR "decubitus ulcer*"[tiab] OR bedsore*[tiab] OR EPUAP[tiab] OR NPIAP[tiab] OR NPUAP[tiab]) AND ("Patient Reported Outcome Measures"[Mesh] OR "Patient Outcome Assessment"[Mesh] OR "Patient Satisfaction"[Mesh] OR "Patient Participation"[Mesh] OR "Quality of Life"[Mesh] OR "patient-reported outcome*"[tiab] OR PROM[tiab] OR PROMs[tiab] OR "patient-reported experience*"[tiab] OR PREM[tiab] OR PREMs[tiab] OR "patient experience*"[tiab] OR "lived experience*"[tiab] OR dignity[tiab] OR "patient involvement"[tiab] OR "patient satisfaction"[tiab]) AND ("Intensive Care Units"[Mesh] OR "Critical Care"[Mesh] OR "Hospitals"[Mesh] OR "Community Health Nursing"[Mesh] OR "Home Care Services"[Mesh] OR "Home Nursing"[Mesh] OR hospital*[tiab] OR "acute care"[tiab] OR "intensive care"[tiab] OR ICU[tiab] OR "community nurs*"[tiab] OR "district nurs*"[tiab] OR "home care"[tiab] OR "home nursing"[tiab] OR "community hospital*"[tiab] OR "primary care"[tiab] OR "family and community nurse*"[tiab] OR "public health nurs*"[tiab])) AND (english[la] OR italian[la]) AND 2014:2025[dp]
Scopus	TITLE-ABS-KEY(("pressure ulcer*" OR "pressure injur*" OR "pressure sore*" OR "decubitus ulcer*" OR bedsore* OR EPUAP OR NPIAP OR NPUAP) AND ("patient-reported outcome*" OR PROM OR PROMs OR "patient-reported experience*" OR PREM OR PREMs OR "patient experience*" OR "lived experience*" OR "quality of life" OR dignity OR "patient satisfaction" OR "patient involvement") AND (hospital* OR "acute care" OR "intensive care" OR ICU OR "community nurs*" OR "district nurs*" OR "home care" OR "home nursing" OR "community hospital*" OR "primary care" OR "family and community nurse*" OR "public health nurs*")) AND PUBYEAR > 2013 AND PUBYEAR < 2026
CINAHL (EBSCOhost)	((MH "Pressure Ulcer+") OR TI ("pressure ulcer*" OR "pressure injur*" OR "decubitus*" OR bedsore* OR EPUAP OR NPIAP OR NPUAP) OR AB ("pressure ulcer*" OR "pressure injur*" OR "decubitus*")) AND ((MH "Patient Reported Outcome Measures") OR (MH "Patient Satisfaction") OR (MH "Quality of Life") OR (MH "Consumer Participation") OR (MH "Patient Centered Care") OR TI ("patient-reported outcome*" OR PROM OR PROMs OR "patient-reported experience*" OR PREM OR PREMs OR "patient experience*" OR dignity) OR AB ("patient-reported" OR PROM OR PREM OR "patient experience*" OR dignity)) AND ((MH "Intensive Care Units+") OR (MH "Community Health Nursing+") OR (MH "Home Health Care+") OR (MH "Hospitals+") OR TI (hospital* OR "intensive care" OR ICU OR "community nurs*" OR "district nurs*" OR "home care" OR "community hospital*" OR "family and community nurse*") OR AB (hospital* OR "community nurs*" OR "home care" OR "district nurs*"))

The strategy combined three thematic blocks with the Boolean operator AND. The first was a pressure injury and staging block, the second a PROMs, PREMs and patient-reported block, and the third a setting block spanning hospital care, including intensive and acute care, and community and home nursing. Synonyms within each block were linked by OR, and controlled vocabulary (MeSH and CINAHL Subject Headings) was paired with free-text terms. European origin was analysed as a distinct lens and supported by a supplementary Europe-restricted run, rather than imposed on the primary search, because combining a Europe block with AND markedly reduces the yield. Table 1 presents the Boolean search strings used in each database, together with the controlled-vocabulary terms and the applied publication year, language and database limits. The strategy was developed for validation by a biomedical librarian prior to the definitive run.

Eligibility Criteria

Sources were eligible if they concerned adult patients (aged 18 years or older) at risk of, or living with, pressure injuries classifiable by EPUAP/NPIAP staging, as defined in the international prevention and treatment guideline and its revised staging system [2,18]. Included patients received nursing care in European hospital settings, comprising acute, intensive and sub-intensive care, or in community settings, comprising community, district and home nursing, community hospitals and community houses. Each eligible source reported a patient-reported dimension, a PROM and/or a PREM, obtained directly or via a validated proxy. Primary studies of any design, systematic and scoping reviews, clinical practice guidelines, policy and normative documents defining the community care framework, and relevant grey literature were eligible, in English or Italian. Sources were excluded if they concerned paediatric populations, reported only clinician-rated or device-measured outcomes with no patient-reported dimension, were editorials, letters or commentaries lacking empirical content, or had a non-European primary focus that contributed nothing to the European lens. A source was classified as European if it was conducted in, or reported data derived from, one or more countries of geographic Europe (WHO European Region). Turkey was treated as transcontinental and retained on a source-by-source basis, because the two Turkish records are Pressure Ulcer Quality of Life (PU-QOL) instrument validation and characterisation studies that inform the instrument-mapping objective rather than the European prevalence picture. Multinational reviews were retained when they included European settings or data, or informed the instrument and domain mapping. Consistent with the mapping rather than appraisal purpose of a scoping review, no fixed minimum proportion of European participants was required; each such source was judged on its contribution to the European analytical lens, with country confirmed from the full text and author affiliations.

The eligibility criteria are summarised in Table 2.

**Table 2:** Eligibility criteria within the Population, Concept and Context framework PCC: Population, Concept, Context, EPUAP/NPIAP: European Pressure Ulcer Advisory Panel/National Pressure Injury Advisory Panel, PROM: patient-reported outcome measure, PREM: patient-reported experience measure

Inclusion	Exclusion
Adults (aged 18 years or older) receiving nursing care in European hospital settings (acute, including intensive and sub-intensive care) or community settings (community, district and home nursing, community hospitals, community houses)	Paediatric populations; non-European primary focus that contributes nothing to the European lens
At risk of, or living with, pressure injuries classifiable by EPUAP/NPIAP staging	Pressure injury not relatable to EPUAP/NPIAP staging, where the stage cannot be inferred
Reports a patient-reported dimension (a PROM and/or a PREM), directly or via a validated proxy	Purely clinician-rated or device-measured outcomes with no patient-reported dimension
Any design; reviews, guidelines, policy and normative documents defining the community care framework, and retrievable grey literature; English or Italian	Editorials, commentaries or opinion pieces (background only); languages not feasible to translate; no retrievable full text

Source Selection

**Figure 1:**
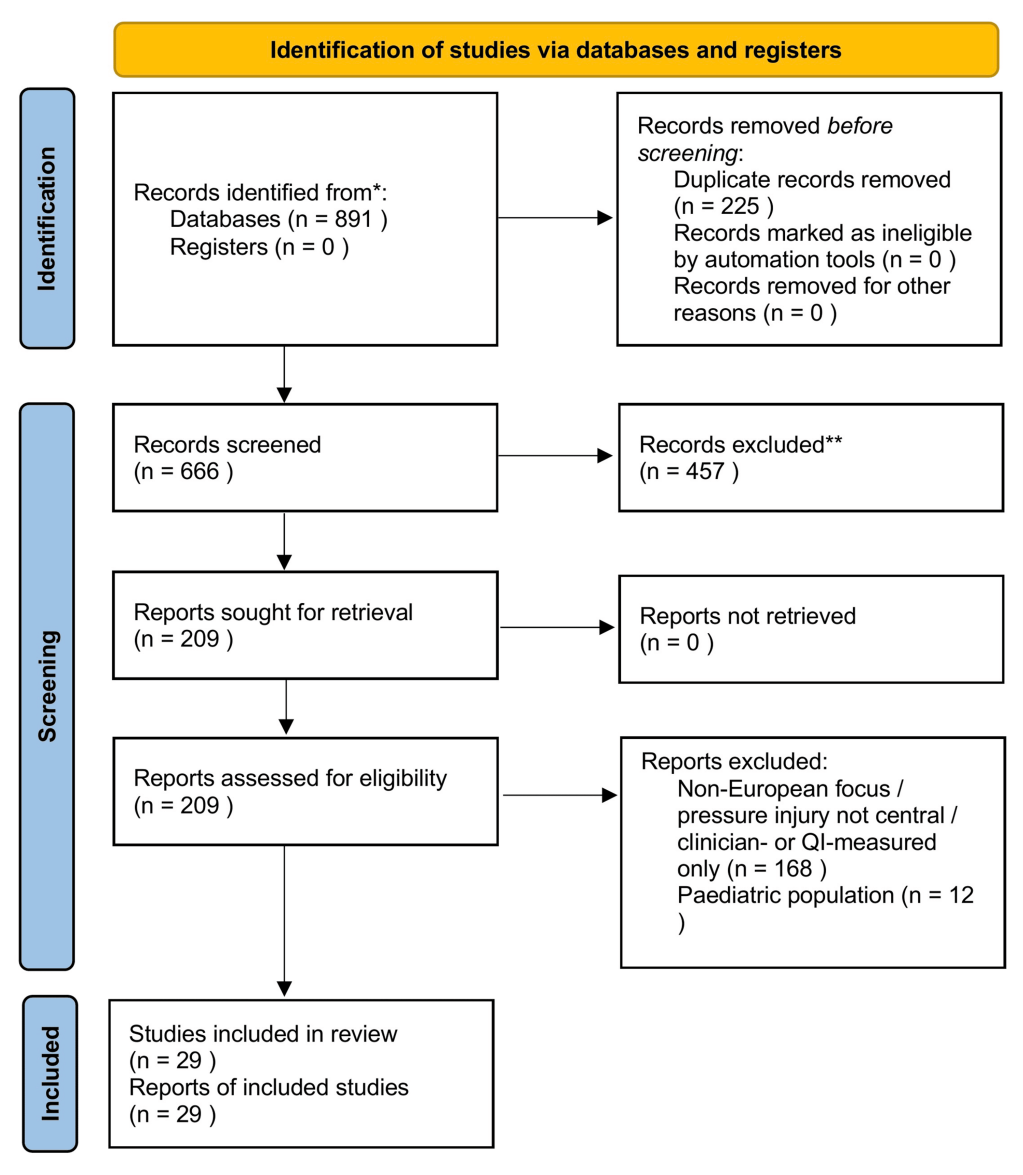
PRISMA 2020 flow diagram for new systematic reviews, which included searches of databases and registers only PRISMA 2020 flow diagram adapted for scoping reviews, illustrating the study selection process. The diagram uses the official PRISMA 2020 template (databases and registers pathway) completed with the review's numbers; the other methods pathway was not used, so its counts are zero. Databases searched: PubMed, Scopus and CINAHL PRISMA: Preferred Reporting Items for Systematic Reviews and Meta-Analyses, CINAHL: Cumulative Index to Nursing and Allied Health Literature

All records retrieved from the three databases were collated and imported into Rayyan [19], where exact duplicates were automatically resolved. Potential duplicates flagged by the software's fuzzy-matching algorithm were manually reviewed and reconciled on the basis of title, authors and publication year, and the unique records were carried forward to screening. Following a calibration exercise, three reviewers (GF, GM and GG) independently screened titles and abstracts for relevance to the PCC question and assessed the full texts against the inclusion and exclusion criteria. Disagreements were resolved by discussion, and a fourth author (VA) acted as arbitrator. Initial title and abstract charting and following full-text check are resolved by reviewers. Grey literature in the form of conference proceedings was captured through the database searches (notably CINAHL) and screened together with all other records, and the Italian normative framework and the international guideline were additionally sought from official sources to inform the Context. The selection process was documented using a PRISMA 2020 flow diagram adapted for scoping reviews and is shown in Figure 1; the numbers of records at each stage, with the reasons for exclusion, are reported in the Results.

Data Extraction and Charting

Data were charted into a predefined form that was piloted and refined before full use. The charting fields comprised the citation and origin (authors, year, country and European setting yes or no); the source type and design; the setting and the professional delivering care; the population and staging system; the pressure injury stage or stages addressed, from prevention and at-risk through stage 1 to 4, unstageable and deep tissue pressure injury; the PROMs used and the domains captured, namely, pain, exudate, sleep, mobility, dignity and psychological impact; the PREMs used and the prevention domains captured, namely, involvement, communication, perception of repositioning and support surfaces, and satisfaction; the named instruments and mode of administration (self-report or validated proxy); whether a dedicated pressure injury protocol or care pathway was reported for the setting; whether and how the patient-reported axis was linked to the objective stage; and the key findings and gaps. Each charted value is traceable to its source record and its DOI or PubMed identifier (PMID); where a dimension was not stated in the source, it was recorded as not reported. The complete study-by-study charting matrix is presented in the Results, and the record-level screening decisions for all 666 records are provided in the accompanying data workbook (Appendices).

Synthesis and Presentation of Results

Consistent with JBI methodology for scoping reviews, the charted data were summarised using a narrative approach supported by tabulation. Findings were organised around the review objectives, namely, the PROMs and PREMs identified, the domains captured at each EPUAP/NPIAP stage and during prevention, the instruments used and their mode of administration, the role of the community and family nurse, and whether dedicated protocols were reported. A conceptual matrix, presented in the Discussion, arranges the patient-reported domains against the staging continuum and the prevention phase, applied separately to hospital and community settings.

Results

Selection of Sources

The three database search returned 891 records, comprising 251 from PubMed, 427 from Scopus and 213 from CINAHL. After removal of 225 duplicates, 666 records were screened by title and abstract, of which 457 were excluded (374 with no patient-reported dimension and 83 with no pressure injury concept). The remaining 209 reports were assessed for eligibility, 180 were excluded (168 with a non-European primary focus, pressure injury not central, or only clinician- or quality improvement-measured outcomes, and 12 concerning paediatric populations) and 29 sources were included. The study selection flow is shown in Figure 1 (Methods), and the excluded records are detailed by reason in Table 3.

**Table 3:** Records excluded during screening and eligibility assessment, with reasons Of the 666 unique records screened, 457 were excluded at title and abstract screening, and 180 of the 209 reports assessed for eligibility were excluded, leaving 29 included sources. The record-level decision and reason for every screened record are provided in the accompanying data workbook (Appendices) (sheet "All_records_screening"). Screening was performed on titles and abstracts and full texts by the review team.

Stage	Reason for exclusion	n
Title-and-abstract screening	No patient-reported dimension	374
Title-and-abstract screening	No pressure injury concept	83
Eligibility assessment	Non-European primary focus, pressure injury not central, or only clinician- or quality-improvement-measured outcomes	168
Eligibility assessment	Paediatric population	12
Total excluded		637

Characteristics of Included Sources

The 29 included sources varied in design, setting and staging system; their aggregate characteristics are presented in Table 4 and the full study-by-study charting in Table 5 [20-48]. Twenty-five were conducted in European settings, two in Turkey and two were multinational reviews. Designs comprised primary quantitative (n = 11) and primary qualitative (n = 9) studies, six mixed-methods studies and three reviews (two scoping and one Cochrane systematic review). Care was delivered in hospital settings (n = 12), in community, district or home nursing (n = 8), or across the hospital-to-home transition (n = 6); no included source was set in an Italian community hospital or community house. A community, district or family nurse was the named provider in four sources, with informal family caregivers in a further two, and the staging system or stage was not stated in 23 of the 29 sources. Of the 29 included sources, 23 were completed empirical studies, three were study protocols and three were evidence syntheses. For the three protocols, the PROMs and PREMs listed were planned instruments rather than administered and reported data [39,43,48].

**Table 4:** Characteristics of included sources All percentages are computed on the 29 included sources (denominator n = 29) within each category (source type, setting, care provider, geographic origin, staging system, patient-reported axis, link to stage and dedicated protocol) and are rounded, so columns may not total exactly 100%. Categories were charted from the full texts by the review team. PI: pressure injury, PROM: patient-reported outcome measure, PREM: patient-reported experience measure, EQ-5D: EuroQol five-dimension, SF-36: 36-Item Short Form, PU-QOL: Pressure Ulcer Quality of Life, QPP: Quality from the Patient's Perspective, EPUAP/NPIAP: European Pressure Ulcer Advisory Panel and National Pressure Injury Advisory Panel

Characteristic	Number (%)
Source type	
Primary quantitative	11 (38%)
Primary qualitative	9 (31%)
Mixed-methods	6 (21%)
Review (scoping or systematic)	3 (10%)
Setting	
Hospital (intensive/acute/ward/rehabilitation)	12 (41%)
Community/district/home nursing	8 (28%)
Hospital-to-home (transitional)	6 (21%)
Not specified	3 (10%)
Community hospital; community house	0 (0%)
Care provider	
Community/district/family nurse	4 (14%)
Hospital or other nurse	6 (21%)
Multidisciplinary	9 (31%)
Informal (family) caregiver	2 (7%)
Not specified	8 (28%)
Geographic origin	
European setting	25 (86%)
Turkey (transcontinental)	2 (7%)
Multinational review	2 (7%)
Staging system	
EPUAP/NPIAP or stage/category stated	6 (21%)
Not stated	23 (79%)
Patient-reported axis	
PROM only	12 (41%)
PREM only	9 (31%)
Both PROM and PREM	5 (17%)
Review (patient-centred)	3 (10%)
Link to EPUAP/NPIAP stage	
Partial	3 (10%)
Absent	26 (90%)
Dedicated protocol reported	
Yes	5 (17%)
No/not reported	24 (83%)

**Table 5:** Study-by-study charting of the 29 included sources of evidence No included source was set in an Italian community hospital or community house. Data were charted from the full texts; "Not reported" denotes an item not stated in the full text. Titles and abstracts were used only for screening. Each row is traceable to its reference and DOI. Ref: reference number in this article (references 20-48, listed in the References section), PROM: patient-reported outcome measure, PREM: patient-reported experience measure, DTPI: deep tissue pressure injury, SCI: spinal cord injury, EQ-5D: EuroQol five-dimension, SF-36: 36-Item Short Form, PU-QOL: Pressure Ulcer Quality of Life, Wound-QoL: Wound Quality of Life, WOUND-Q: chronic-wound patient-reported outcome measure under development, QPP: Quality from the Patient's Perspective, PURPOSE T: Pressure Ulcer Risk Primary Or Secondary Evaluation Tool, ICU: intensive care unit, RCT: randomized controlled trial, QALYs: quality-adjusted life years, SCI: spinal cord injury, PI: pressure injury, GP: general practitioner, ICT: information and communications technology, VAS: visual analogue scale, JBI: Joanna Briggs Institute

Ref	Source (first author, year)	Country	Setting; provider	Design; staging	Stage(s)	Protocol	PROM/PREM: domains; instrument; mode	Linkage; key finding
[20]	Binda et al. (2026)	Italy	Hospital (ICU/post-ICU); not specified	Prospective observational; NPIAP	With injury (heel PU)	No	PROM: mobility/function; HRQoL; EQ-5D-5L; Barthel; Manchester Mobility Score; self-report	No; HRQoL and mobility impaired 1 year after ICU discharge in survivors with heel PU
[21]	Irgens (2025)	Norway	Hospital and community (district nursing); community/family nurse (district nurse)	Mixed methods (prevalence + qualitative); not reported (abstract)	Prevention and with injury	Not reported	PREM/PROM: lived experience; psychological (isolation); continuity of care; semi-structured interviews; thematic analysis; self-report + family/proxy	No; patients described reduced quality of life from isolation and inconsistent care; digital continuity valued
[22]	Irgens et al. (2024)	Norway	Hospital (spinal cord units) + home (videoconference); multidisciplinary	RCT with cost-utility analysis; not reported (abstract)	With injury	Not reported	PROM: HRQoL (QALYs); EQ-5D-5L; self-report	No; videoconference added to regular care assessed via QALYs (EQ-5D-5L) in patients with SCI with PI
[23]	Zanini et al. (2020)	Switzerland	Community (community-dwelling); not specified	Qualitative (thematic analysis); not reported (abstract)	Prevention/at-risk	Not reported	PREM: involvement; self-management; prevention behaviours; semi-structured interviews; self-report	No; community-dwelling individuals with SCI adopt different "styles" of PI prevention
[24]	Zanini et al. (2020)	Switzerland	Community/outpatient; community/family nurse; GP; multidisciplinary	Qualitative (thematic analysis); not reported (abstract)	Prevention/at-risk	No (fragmented care)	PREM: experience of prevention; continuity; unmet needs; semi-structured interviews; self-report + family/proxy	No; outpatient/community PI prevention for SCI is fragmented and mono-professional; needs unmet
[25]	García-Sánchez et al. (2019)	Spain	Community (home care); community/primary care	Qualitative (grounded theory); not reported (abstract)	With injury	Not reported	PREM: experience/perceptions of home PU care; caregiver views; in-depth interviews; self-report + family/proxy	No; patients and caregivers conceptualise home care as the preferred approach for PU
[26]	García-Sánchez et al. (2019)	Spain	Community (home care); primary care services	Qualitative (grounded theory); not reported (abstract)	With injury	Not reported	PREM: caregiver involvement; barriers/facilitators; satisfaction with care; interviews; family/proxy (caregivers)	No; barriers/facilitators to home caregiver involvement in PI care identified
[27]	Hultin et al. (2019)	Sweden	Hospital (orthopaedic rehabilitation); hospital nurse	Qualitative descriptive; not reported (abstract)	Prevention/at-risk	Not reported	PREM: involvement/participation; awareness; communication; semi-structured interviews; self-report	No; ICT (bedside pressure mapping) increased older patients' participation in PI prevention
[28]	Bååth et al. (2024)	Sweden	Community (home/outpatient, mobile PU team); multidisciplinary (mobile PU team)	Cross-sectional; modified Norton; category 4	With injury (deep, category 4)	Yes (mobile PU team pathway)	PROM/PREM: satisfaction/experience of care; wound-related QoL; QPP; Wound-QoL; m Norton; self-report	No; home/outpatient mobile team care for deep PU; patient-reported quality and Wound-QoL measured
[29]	Hultin et al. (2023)	Sweden	Hospital ward; hospital nurse	Mixed methods (feasibility); stages I-IV (risk tool)	Prevention/at-risk	Not reported	PREM: participation; experience of risk assessment; patient interviews; nurse focus groups (PURPOSE T); self-report	No; feasibility of the electronic PURPOSE T pressure ulcer risk instrument in a Swedish hospital ward; patients were interviewed on their participation in prevention
[30]	Skogestad et al. (2017)	Norway	Hospital (acute medical wards); hospital nurse	Prospective cross-sectional survey; stages I-IV (EPUAP type)	Prevention/at-risk	Not reported	PROM: pain; symptom occurrence/distress; Memorial Symptom Assessment Scale; pain NRS; Braden; self-report	Partial (symptoms versus Braden risk); self-reported symptoms/distress add to Braden for PU risk in medical inpatients
[31]	Nixon et al. (2015)	United Kingdom	Hospital (acute) and community; multidisciplinary	Programme of research (mixed methods, psychometrics); EPUAP/NPUAP	Prevention/at-risk and with injury	Yes (risk assessment framework developed)	PROM: pain; HRQoL; health utility; patient involvement; PU-QOL and Health Utility measures (developed); pain prevalence; self-report	Partial; PURPOSE programme developed pressure ulcer PROMs (PU-QOL) and a risk assessment framework in the UK
[32]	Essex et al. (2009)	United Kingdom	Hospital (inpatients); not specified	Cohort + pilot; not reported (abstract)	With injury	No	PROM: HRQoL (physical/mental); pain; SF-36; EQ-5D; pain VAS; self-report	No; inpatients with PU had significantly lower SF-36 physical/mental scores than those without
[33]	Spilsbury et al. (2007)	United Kingdom	Hospital (inpatients); not specified	Qualitative (semi-structured interviews); not reported (abstract)	With injury	Not reported (abstract)	PROM/PREM: lived experience; QoL impact; pain; dignity; semi-structured interviews; self-report	No; hospital inpatients describe substantial impact of PU and its treatment on health and quality of life
[34]	Jackson et al. (2017)	United Kingdom	Community (district nursing); community/family nurse (district nurse)	Mixed methods (case study); not reported (abstract)	With injury	Not reported	PREM: involvement; experience of/continuity of care; support-surface use; semi-structured interviews (COREQ); self-report	No; community PI patients felt reliant on services and concerned about care consistency; authentic involvement needed
[35]	Reeves et al. (2025)	United Kingdom	Hospital (secondary) and primary care; multidisciplinary (including GPs, nurses)	Mixed methods (systematic reviews, cohort, survey, consensus); severe (category 3-4)	With injury (severe)	Not reported	PROM: HRQoL impact of severe PU; HRQoL review; surveys; self-report	Partial; SIPS feasibility study of surgical reconstruction for severe PU, including HRQoL impact review
[36]	Dal Molin et al. (2018)	Italy	Hospital (inpatient units); hospital nurse (primary nursing)	Before-after; not reported (abstract)	Prevention/at-risk	Not reported	PREM: satisfaction with care; patient satisfaction (subgroup); self-report	No; primary-nursing model; PU among nursing-sensitive outcomes; patient satisfaction collected in a subgroup
[37]	Gül et al. (2023)	Turkey	Hospital (private/state/university); wound-care nursing	Methodological (validation); with injury (PI)	With injury	Not reported	PROM: PU-specific QoL (mobility, pain, sleep, exudate, odour); PU-QOL - Turkish; WHOQOL-BREF; self-report	No; Turkish PU-QOL adaptation shows acceptable validity/reliability (Rasch)
[38]	Gül et al. (2025)	Turkey	Hospital (private/state); not specified	Descriptive/correlational; with injury (PI)	With injury	Not reported	PROM: PU-specific QoL (mobility, activity, pain, sleep, odour, exudate); PU-QOL - Turkish; self-report	No; worst QoL domains were mobility, activity, pain, sleep, odour, exudate; more injuries/longer healing worsened QoL
[39]	Klassen et al. (2020)	Denmark, Netherlands, Canada and USA	Mixed (chronic wounds, including PU); not specified	Mixed methods PROM development protocol; not reported (abstract)	With injury (chronic wounds, including PU)	Not reported	PROM: patient-important wound outcomes (multi-domain); WOUND-Q (developed); self-report	No; international development of WOUND-Q, a PROM for all chronic wound types including pressure ulcers (European sites included)
[40]	Soegaard et al. (2023)	Denmark	Community / hospital (SCI); not specified	Qualitative (phenomenological-hermeneutic); not reported (abstract)	With injury	No (incoherent treatment)	PROM/PREM: lived experience; balancing life versus prevention; QoL; semi-structured in-depth interviews; self-report	No; people with SCI describe prolonged, incoherent pressure ulcer treatment that puts an active life "on hold" (conference version of Soegaard et al., J Tissue Viability 2024)
[41]	Riaz et al. (2026)	Multinational (review)	Hospital (acute care); multidisciplinary	Scoping review; not reported (abstract)	Prevention and with injury	Not reported	PREM (review): patient-centred approaches; involvement; JBI scoping review; n/a (review)	No; scoping review mapping patient-centred approaches to PI prevention/management in adult acute care
[42]	Soegaard et al. (2023)	Denmark and Belgium	Transitional (hospital to home); multidisciplinary	Scoping review; not reported (abstract)	Prevention/at-risk	Not reported	PREM (review): stakeholder perspectives; organisation of prevention; JBI scoping review; n/a (review)	No; scoping review of PU prevention for SCI in transition from hospital/rehab to home
[43]	Richards et al. (2021)	United Kingdom	Hospital (acute, SARS-CoV-2 wards); hospital nurse	Cluster RCT (protocol); not reported (abstract)	Prevention/at-risk	Yes (fundamental nursing care protocol)	PREM/PROM: experience of care; involvement; relational care; psychological; HRQoL; PI as care quality outcome; QPP; Relational Aspects of Care Questionnaire; EQ-5D; PHQ-2; GAD-2; self-report	No; cluster RCT protocol testing whether a fundamental nursing care protocol improves patient-reported experience; pressure injuries are a secondary care quality outcome
[44]	O'Connor et al. (2021)	Multinational (review)	Any care setting; multidisciplinary	Cochrane systematic review; not reported (abstract)	Prevention/at-risk	Not reported	PREM (review): patient and carer education; involvement in prevention; systematic review of RCTs; n/a (review)	No; cochrane review of patient and/or lay-carer education for preventing pressure ulceration in at-risk people
[45]	Robineau et al. (2019)	France	Hospital/outpatient (SCI); multidisciplinary	Prospective interventional (pre-post); not reported (abstract)	Prevention/at-risk	Yes (patient education programme)	PROM: HRQoL; self-esteem; self-efficacy; psychological; skin self-management; SF-36; Rosenberg Self-Esteem Scale; Schwarzer self-efficacy; Hospital Anxiety and Depression Scale; Revised SMnac; self-report	No; a patient education programme significantly improved skin self-management in adults with chronic SCI at risk of pressure ulcers
[46]	van Gaal et al. (2022)	Netherlands	Community; multidisciplinary; informal caregivers	Qualitative (deductive thematic analysis); with injury	Prevention and with injury	No	PREM: self-management experience; involvement; peer support; social impact; semi-structured interviews; self-report + family/proxy	No; adults with SCI self-manage pressure ulcer prevention and treatment through tailored measures, peer support and reliance on informal caregivers
[47]	Rodrigues et al. (2016)	Portugal and Spain	Community (home care); informal (family) caregivers	Descriptive/correlational; with injury	With injury	Not reported	PROM (proxy/caregiver): Caregiver HRQoL; caregiver burden; SF-36v2; Burden Interview Scale; family/proxy (caregivers)	No; informal caregivers of patients with pressure ulcer at home report low quality of life and significant burden, worsening with more ulcers and direct wound care
[48]	Irgens et al. (2019)	Norway	Hospital and home (telemedicine); multidisciplinary	Mixed epidemiological + RCT (protocol); with injury (healing monitored)	Prevention and with injury	Yes (teleSCI follow-up)	PROM: HRQoL; wound healing; need for assistance; EQ-5D; TIME registration; Photographic Wound Assessment Tool; self-report	No; protocol comparing in-hospital versus home telemedicine follow-up of pressure ulcers in SCI, with HRQoL (EQ-5D) outcomes

PROMs Across the Staging Continuum

Patient-reported outcomes were reported in 17 sources [20-22,28,30-33,35,37-40,43,45,47-48], 12 as the sole patient-reported axis and 5 alongside a PREM. The instruments identified were the generic EuroQol five-dimension (EQ-5D and EQ-5D-5L) and 36-Item Short Form (SF-36) health surveys, the pressure ulcer-specific Pressure Ulcer Quality of Life (PU-QOL) instrument and the Wound Quality of Life (Wound-QoL) measure, the multi-wound WOUND-Q measure under development, and, for symptoms, the Memorial Symptom Assessment Scale and pain numeric rating and visual analogue scales. The domains captured were pain, mobility and function, sleep, exudate and odour, dignity, psychological impact and overall health-related quality of life. As shown in Table 6, these domains were captured for established injury in the aggregate but were rarely resolved to a specific EPUAP/NPIAP stage.

**Table 6:** Conceptual matrix: patient-reported domains across the EPUAP/NPIAP staging continuum and the prevention phase Yes: domain captured, Aggregate: captured in the charted evidence, but not resolved to an individual EPUAP/NPIAP stage, No: absent No included source resolved any domain to a single stage, so the former per-stage columns have been collapsed into one "established pressure injury (stage not resolved)" column. The pattern was consistent across hospital and community settings; cells were charted from the full texts by the review team. EPUAP/NPIAP: European Pressure Ulcer Advisory Panel/National Pressure Injury Advisory Panel

Patient-reported domain	Prevention/at-risk	Established pressure injury (stage not resolved)
PROM - Pain	No	Aggregate
PROM - Exudate/wound symptoms	No	Aggregate
PROM - Sleep	No	Aggregate
PROM - Mobility/function	Aggregate	Aggregate
PROM - Dignity	No	Aggregate
PROM - Psychological impact	Aggregate	Aggregate
PREM - Involvement in decisions	Yes	No
PREM - Communication	Yes	No
PREM - Repositioning/support surface perception	Aggregate	No
PREM - Satisfaction with preventive care	Yes	Aggregate

PREMs and the Lived Experience of Prevention

Patient-reported experiences were reported in 14 sources [21,23-29,33-34,36,40,43,46], 9 as the sole patient-reported axis and 5 alongside a PROM, most often through semi-structured interviews and, quantitatively, the Quality from the Patient's Perspective (QPP) instrument and patient satisfaction and patient participation surveys. The prevention domains captured were involvement in care, communication and continuity, the perception of repositioning and support surfaces, and satisfaction with preventive care. These experience domains were concentrated in the prevention and at-risk phase, particularly in community, district and home nursing.

Community and Family Nurse Settings, and Dedicated Protocols

Fourteen sources originated from community, district or home nursing or from the hospital-to-home transition, and a community, district or family nurse was the named care provider in four, with informal family caregivers in a further two. Qualitative studies from Norway, Switzerland, Spain, Sweden, the Netherlands and the United Kingdom [21,23-26,34,46] described the district or community nurse as central to prevention, continuity and support surface management, while patients and family caregivers reported fragmented care and concern about its consistency. No included source was set in the Italian community hospitals (ospedali di comunità) or community houses (case di comunità), and a dedicated pressure injury protocol, care pathway or structured programme was reported in only five sources, including a Swedish mobile pressure ulcer team, the United Kingdom PURPOSE risk assessment framework, and structured patient education and telemedicine follow-up programmes [28,31,43,45,48]. No dedicated protocols or standardised patient-reported measurement systems were identified in the literature searched for these structures; this is an evidence gap in the reviewed literature rather than a confirmed absence in clinical practice.

Linkage to Staging, Instruments and European Evidence

Few sources connected patient-reported data to the objective stage of tissue damage. The staging system or stage was not stated in 23 of the 29 sources, and an explicit link between a PROM or PREM and a specific EPUAP/NPIAP stage was absent in 26 sources and only partial in 3. The named instruments spanned generic HRQoL measures (EQ-5D and SF-36), pressure ulcer-specific measures (PU-QOL, Wound-QoL and WOUND-Q) and an experience measure (QPP), administered by self-report and, for community and end-of-life care, by family or proxy report. The European evidence was concentrated in the United Kingdom, the Nordic countries, Switzerland, Spain and Italy, with two transcontinental Turkish PU-QOL studies [37,38] and one multinational instrument-development study [39] contributing to the analytical lens. Three reviews synthesised the broader evidence on patient-centred approaches to prevention and management, on the organisation of transitional care, and on patient and carer education [41,42,44].

Discussion

This scoping review mapped how the patient's experience of pressure injuries and of their prevention is captured across the EPUAP/NPIAP staging continuum in European hospital and community nursing care. Across the 29 included sources, the patient-reported axis was captured with a small and heterogeneous set of instruments, namely, the generic EQ-5D and SF-36 health surveys, the pressure ulcer-specific PU-QOL and Wound-QoL measures, the WOUND-Q measure under development and the QPP experience measure, supplemented by qualitative interviews. Outcome domains such as pain, mobility, sleep, exudate and health-related quality of life were captured for established injury, whereas experience domains such as involvement, communication and satisfaction were concentrated in the prevention phase. A link to a specific EPUAP/NPIAP stage was absent or only partial in almost every source. This is consistent with the expectation from the background literature that tissue-level staging and the patient-reported axis remain only loosely connected, and that the connection is weakest as care crosses from hospital to community [4,6].

The conceptual contribution of this review is the domain-by-stage matrix (Table 6), which arranges PROM and PREM domains against the prevention phase and each EPUAP/NPIAP stage, marking each cell as captured, partially captured or absent in the charted evidence. Applied separately to hospital and community settings, the matrix makes explicit not only where the patient-reported axis is missing but also where it disappears as the patient moves along the care pathway.

A specific and policy-relevant question concerns whether dedicated protocols exist for the assessment, prevention and management of patients with pressure injuries in the community intermediate care structures introduced by DM 77/2022 and staffed by the family and community nurse [49,50]. No included source was set in these structures, and a dedicated pressure injury protocol, pathway or programme was reported in only five of the 29 sources. Given the recent establishment and heterogeneous regional implementation of the structures [9,10], this dimension emerges as an evidence gap: no dedicated protocols or standardised patient-reported measurement systems were identified in the literature searched for these structures. Whether such protocols exist in current practice cannot be determined from this review. Comparable gaps may be present in district and home nursing services abroad.

The two ends of the continuum challenge measurement in different ways. In high-acuity hospital care, sedation and mechanical ventilation frequently preclude self-report, so proxy-reported and recovery phase data may predominate. In community and home care, self-report is feasible but rarely standardised. Prevention, by contrast, is a continuous and guideline-mandated nursing activity in both settings, in which patients and families can often be engaged. This makes PREMs a tractable near-term target [3,8].

Conceptual Matrix

Table 6 presents the analytical device of the review. It maps the patient-reported domains, shown as rows, across the prevention phase and each EPUAP/NPIAP stage, shown as columns. In the charted evidence, experience (PREM) domains were captured mainly during prevention, whereas outcome (PROM) domains were captured for established injury without being resolved to an individual stage.

Limitations

This review has several limitations, and each was anticipated and, as far as possible, mitigated. First, no single controlled vocabulary term links pressure injury staging to the patient-reported axis. The search therefore combined subject headings with free-text synonyms across three concept blocks, with the terms in each block linked by OR. This design favours sensitivity over precision and was developed for validation by a biomedical librarian before the definitive run. Despite the broad search strategy, the possibility that some relevant sources were missed cannot be excluded; this is consistent with the mapping purpose of a scoping review, which charts the extent and nature of the evidence rather than retrieving every record exhaustively.

Second, three databases were searched, namely, PubMed, Scopus and CINAHL, and Web of Science and Embase were not. The databases chosen have complementary coverage. CINAHL is the principal database for the nursing and allied health literature that is central to this review, and Scopus overlaps substantially with Web of Science and Embase in the health sciences. The practical loss of coverage is therefore smaller than the number of databases suggests.

Third, because the acronym EPUAP contains the word European, country of origin could not be used as a reliable title and abstract filter. A brittle geographic filter might have excluded eligible European studies. European origin was therefore treated as an analytical lens, and country was confirmed from the full text and the author affiliations during eligibility assessment. This approach is more sensitive than a hard filter and protects against the wrongful exclusion of relevant records. The record-level decisions are reported in the accompanying workbook (Appendices), so the process is transparent and reproducible, and accountability rests with the review team.

Fourth, records were restricted to English and Italian. This restriction was deliberate and aligned with the review's aims. The context of interest is the Italian community nurse and intermediate care framework, and English is the dominant language of the international evidence. Italian-language grey literature and normative documents were sought specifically to capture that context, which offsets the restriction for the questions the review set out to answer.

Fifth, consistent with scoping review methodology, no critical appraisal of methodological quality was undertaken. This is a feature of the chosen method rather than an oversight. JBI guidance and the PRISMA-ScR extension state that a scoping review maps the extent and nature of the available evidence rather than weighing its quality. Formal appraisal therefore falls outside its scope, and its absence does not undermine the findings.

## Conclusions

Pressure injury care spans hospital and community nursing, and this scoping review mapped which patient-reported outcome measures and patient-reported experience measures are used across the European Pressure Ulcer Advisory Panel and National Pressure Injury Advisory Panel staging continuum in European hospital and community settings. Twenty-nine sources were included. The patient's experience of pressure injuries and of their prevention is captured with a small and heterogeneous set of instruments and qualitative methods, dominated by the generic EuroQol five-dimension and 36-Item Short Form health surveys and the pressure ulcer-specific Pressure Ulcer Quality of Life, Wound Quality of Life and WOUND-Q measures, and it is rarely connected to the objective stage. Experience measures cluster in the prevention phase and in community, district and home nursing, whereas the link to a specific stage of tissue damage is almost always absent. No included source addressed the new Italian community hospitals or community houses, and no dedicated protocols or standardised patient-reported measurement systems for these structures were identified in the literature searched.

Building on this map, the review proposes a structured research and practice agenda. First, stage-aware patient-reported outcome and experience instruments could be developed and validated for the pressure injury pathway, including versions suitable for critically ill patients, for validated proxy or family respondents, and for community and home use. Second, the patient-reported axis could be embedded in the Italian territorial care framework, since the family and community nurse and the community hospitals and community houses provide the organisational vehicle, whereas the current normative guidance for the role does not yet specify standardised patient-reported measurement. Third, dedicated pressure injury protocols for these intermediate care structures could incorporate patient-reported endpoints alongside the objective stage of tissue damage. Fourth, these instruments and protocols would benefit from prospective testing in European hospital and community nursing settings, with attention to the hospital-to-home transition where the patient-reported axis appeared weakest, and this agenda is offered as a constructive direction for research and policy.

## Appendices

The data workbook containing the record-level screening decisions is available (view-only) at https://docs.google.com/spreadsheets/d/1SRJ0SbBAB-Msioi6mnEuvKETTB2Wziwt/edit?usp=sharing.
