# Correction: Patient-Reported Outcome and Experience Measures (PROMs and PREMs) in Pressure Injury Prevention and Care Across European Hospital and Community Nursing Settings: A Scoping Review

**DOI:** 10.7759/cureus.c468

**Published:** 2026-08-05

**Authors:** Vincenzo Abbasciano, Giuseppe Fumai, Giorgia Chetta, Gianluca Laricchia, Verdiana La Grotta, Giovanna Muto, Giuseppe Gisario, Carla Recchia, Nunzia Cappucci, Valeria Liso, Anna Maria Carrozzo

**Affiliations:** 1 Department of Internal Medicine, Hospital, Local Health Authority Barletta-Andria-Trani, Andria, ITA; 2 Biomedical Sciences, University of Foggia, Foggia, ITA; 3 Orthopedic and Neurological Rehabilitation, Rehcura Rehabilitation Facility, Adelfia, ITA; 4 Stakeholder Engagement and Innovation Governance, Agenzia Regionale Sanitaria della Puglia, Bari, ITA; 5 Nursing, University of Foggia, Foggia, ITA; 6 Information, Communication, University Liaison, and Training, Local Health Authority of Barletta Andria Trani, Barletta, ITA; 7 Cardiothoracic Surgery, University of Padua, Padua, ITA; 8 Emergency, Local Health Authority Foggia, Cerignola, ITA; 9 Complex Administrative-Legal Framework, Local Health Authority Foggia, Foggia, ITA; 10 Department of Orthopedics and Traumatology, Local Health Authority Barletta-Andria-Trani, Andria, ITA; 11 Local Health Authority, University of Foggia, Foggia, ITA

This article has been corrected to update the affiliations for Dr. Vincenzo Abbasciano and Dr. Valeria Liso as Department of Internal Medicine, Hospital, Local Health Authority Barletta-Andria-Trani, Andria, IT, and Department of Orthopedics and Traumatology, Local Health Authority Barletta-Andria-Trani, Andria, IT, respectively.

